# Multiband and multifunctional polarization converter using an asymmetric metasurface

**DOI:** 10.1038/s41598-021-88771-x

**Published:** 2021-04-29

**Authors:** Negin Pouyanfar, Javad Nourinia, Changiz Ghobadi

**Affiliations:** Department of Electrical Engineering, Urmia University, Urmia, Iran

**Keywords:** Engineering, Electrical and electronic engineering

## Abstract

A compact and asymmetric multi-band reflective polarization converter metasurface has been offered in this paper. The proposed simple converter can effectively convert an incident linearly polarized EM wave to its orthogonal counterpart and circular polarized waves (RHCP and LHCP) at two frequency bands. The design consists of a square with two curves on the top right and lower left corners and a square Split Ring Resonator (SRR) responsible for linear-to-linear and linear-to-circular polarization conversions, respectively. The simulated results show that the converter successfully transforms a *y*-polarized incident wave to its orthogonal counterpart in a frequency range of 15.5–16.5 GHz with unity conversion at 16 GHz and circularly-polarized (RHCP) wave at 13 GHz and (LHCP) at 18 GHz, verified through the fabricated and measured sample. Wide angular stability up to 60° oblique incidence along with high efficiency reveals the good applicability of the structure. Moreover, the root cause of the cross-polarization conversion has been analyzed and confirmed through Bi-Mode Foster equivalent circuit and surface current distribution as well. Finally, a fabricated prototype is tested to validate the simulated results through measurement.

## Introduction

Polarization state of the EM wave and its manipulation has been of great importance as well as frequency and phase for various applications in microwave, terahertz and optical frequency ranges^1–3^. Conventional techniques for polarization manipulation specially in optical crystals, require large bulky structures when compared to operating wavelength. Converters’ applications include a vast variety of antenna design, radio communication, radar technology and etc. Several polarization conversion methods have been introduced which suffer from narrow bandwidth, high loss, volumetric structures and angular dependent responses. Due to their unusual property, not easily found in nature, metamaterials can obtain unprecedented opportunities in different fields^4–6^. One of their most common applications is EM polarization control. Therefore, different anisotropic metamaterial structures and chiral metamaterials have been introduced from microwave to optics frequency range^7–10^. However, as previously mentioned their narrow bandwidth generally restricts their applications. It has been certified that metasurfaces^11–13^, as 2-dimentional and planar versions of metamaterials can profoundly control polarization condition of the EM waves. Therefore, to overcome the aforementioned deficiencies, various polarization converters based on metasurfaces have been reported due to their superior properties such as compact size and easy integration with other devices. A novel design of a cross converter has been presented in^14^ which comprises of a modified square patch, bi-layered substrate and a defected ground plane to expand the bandwidth. Several reflection and transmission type polarization converters have been investigated to transform linear or circular polarized waves to their orthogonal counterparts after transmitting or reflecting from the metasurface. Linear-to-linear^15–17^, circular-to-circular/linear^18,19^ and linear-to-circular^20,21^ polarization converters are among the reported literature. Double U-shaped patches^22^, H-shaped patches^23^, Split Ring Resonator^7^ and other shapes metasurface-based structures have been investigated to achieve wideband or multiband polarization conversions. In^24^, an ultra-wideband ellipse shaped LTC converter metasurface is introduced to reach a wideband conversion with wide angular stability. Recently, an oval-shaped cross polarization converter metasurface is investigated to convert a linearly/circularly polarized EM wave to its cross component in the frequency range of 10.2–20.5 GHz with high efficiency and wide angular stability^25^. A reconfigurable butterfly-shaped metasurface is also realized based on active metasurfaces in^26^ in which the structure can be switched between linear and circular polarization conversions under normal incidence. A three-layer, dual-band reflective polarization converter metasurface is reported in^27^ to change the linearly polarized incident wave to its’ cross component. A transmission type polarizer has been offered, to reach linear and circular polarization conversions, but with large angular stability just for LTC conversion^28^. A multiband transmissive converter metasurface based on FSS with SRR unitcell can reach up to 25° angular stability as reported in^29^. Other similar transmission polarization converters have also been investigated with up to 45° robustness to the oblique incident wave^30^. Generally, most of the reported works are single functional, it means that the designed structure can only control and change just one type of polarization in a single band: cross conversion, LTC or CPC conversion. On the other hand, most of the introduced structures operate well only under normal incident EM waves or are in transmissive mode. Recently, both linear and circular polarization conversions were realized through a single multifunctional design. A reflection type polarization rotator metasurface composed of two meander lines and a microstrip line is presented in^31^ to reach both linear and circular conversions under normal incidence at low and high frequencies, respectively. In^32^, an anisotropic reflective metasurface for linear and circular polarization conversions is introduced at three frequency bands with up to 45° angular stability. A most recent study introduces a multi-band and multi-functional reflective metasurface for C, X and K band Applications^33^ providing wide angular stability up to 75° verified through surface current distribution. In addition to polarization conversion feature, a kind of meta-mirror is presented in several studies where the CP handedness is protected^34,35^. The meta-mirror performance in the range of 4.5–6.5 GHz has been obtained utilizing circular SRR^35^. Likewise, a bi-layer meta-mirror has been presented for mid-infrared frequency regime. Various applications realized by meta-mirrors are reported^36^. Generally, most of the single layer multifunctional polarization converters operate in transmission mode and less effort has been conducted to realize multifunctional property for converters with a single layer structure in reflection mode. In this study, a compact, simple and single layer structure of an asymmetric reflection type polarization converter metasurface is fully discussed. It contains a simple SRR and a square with two curves on the top right and lower left corners. The reflection polarization converter is able to convert the incident EM wave to its orthogonal counterpart at 15.5–16.5 GHz frequency band and to a right-hand and left-handed circular polarized wave at 13 GHz and 18 GHz, respectively for *x*- and *y*-polarized incidences. Deep analysis based on the Bi-Mode Foster equivalent circuit model and also surface current distribution have been provided to profoundly understand the structure performance mechanism. The simulation and measurement have been carried out to confirm the work principles of the converter.

## Structure design procedure

**Figure 1:**
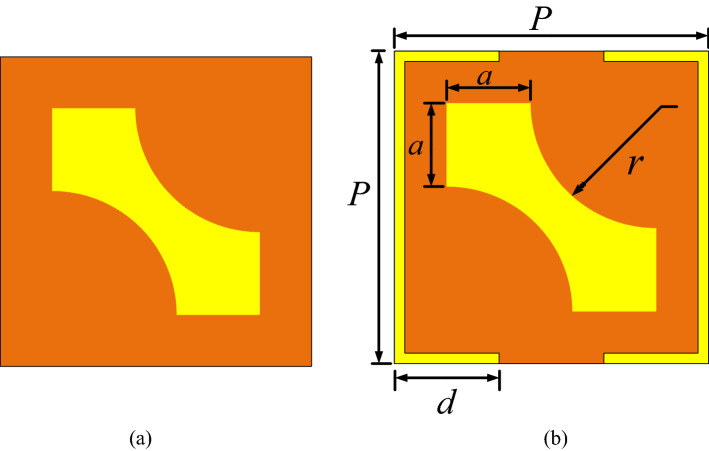
Geometry of the proposed single layer converter, (**a**) without SRR, (**b**) with SRR (figures are extracted from CST microwave Studio software version 2018).

Geometry of the proposed multifunctional converter unit cell is depicted in Fig. 1. The design procedure consists of three parts: a top textured metallic surface, a middle dielectric substrate and a bottom metallic ground. When an EM wave illuminates the structure with a specified polarization, *x*- and *y*-polarized transmitted and reflected EM waves are produced. As a result of multiple reflections between these transmitted EM waves and the metallic ground plane, the final reflected wave is generated. Moreover, reflected EM phase and magnitude can be explicitly controlled through wave interactions into the dielectric and ground plane. Therefore, it is important to choose the structure parameters to avoid bulky and large structure. Hence, since the multiple reflections occur in the dielectric spacer, the thickness should be selected carefully. The top and bottom layers are separated by a FR4 substrate with 4.4 dielectric constant, dielectric loss tangent of 0.02 and thickness of *h*. The copper is used on the top and bottom layers by electric conductivity of *σ* = 5.8 × 10^7^ S/m and 0.035 mm thickness. The design process has been started by a simple square with two curves cut on two sides with radius ‘*r*’ and then a square SRR has been added around it to realize both linear-to-linear and linear-to-circular polarized waves, respectively. This unitcell pattern is wisely selected among different types of the shapes to simply lead to a desired result, so that by employing a modified 45° microstrip and a simple square SRR multifunctional property can be achieved in reflection mode. A single unitcell is simulated with periodic boundary condition along *x*- and *y*-directions using EM software CST Microwave Studio to obtain the cross-polarization conversion in the frequency range of 8–20 GHz. Also, Floquet ports are applied to the periodic structure to study the reflection characteristics. After careful parametric study in CST, final parameters of the unitcell are selected as follows: *p* = 6 mm, *a* = 1.9 mm, *r* = 2.1 mm, *d* = 2 mm, *h* = 2 mm.

As the presented structure is backed by a grounded plane, the reflection characteristic is needed to be examined. Considering a *y*-polarized incident wave, ${r}_{yy}={E}_{y}^{r}/{E}_{y}^{i}$ and ${r}_{xy}={E}_{x}^{r}/{E}_{y}^{i}$ are defined as co-polarization (*y* to *y*) and cross-polarization (*y* to *x*), respectively. Generally, the reflected fields are related to incident ones through Jones matrix as stated in Eq. (1)

1 $$\left[\begin{array}{l}{E}_{x}^{r}\\ {E}_{y}^{r}\end{array}\right]=\left[\begin{array}{ll}{r}_{xx}& {r}_{xy}\\ {r}_{yx}& {r}_{yy}\end{array}\right]\left[\begin{array}{l}{E}_{x}^{i}\\ {E}_{y}^{i}\end{array}\right]$$

**Figure 2:**
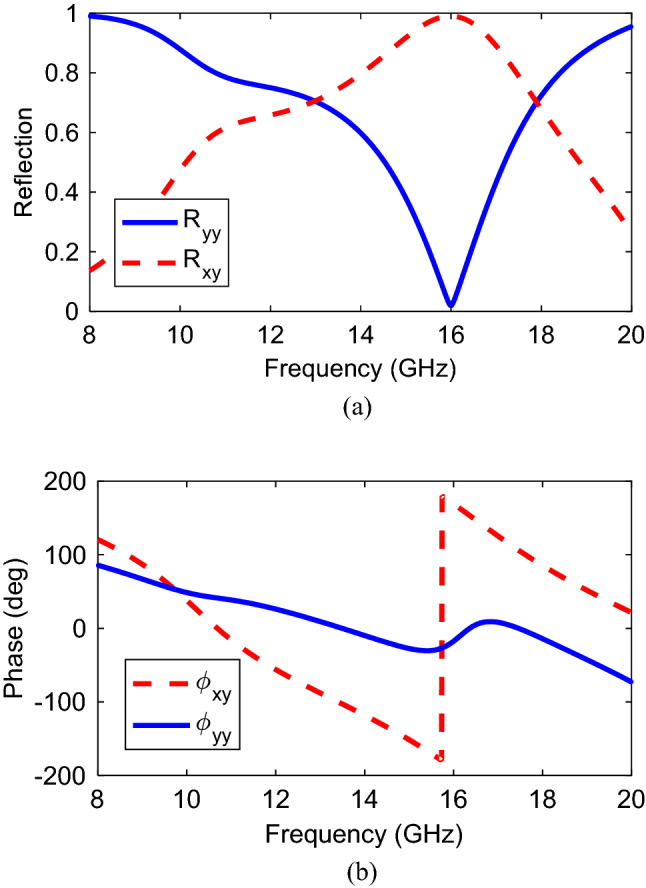
Co- and cross- polarized reflection coefficient for *y*-polarized incident wave (**a**) magnitude, (**b**) phase.

**Figure 3:**
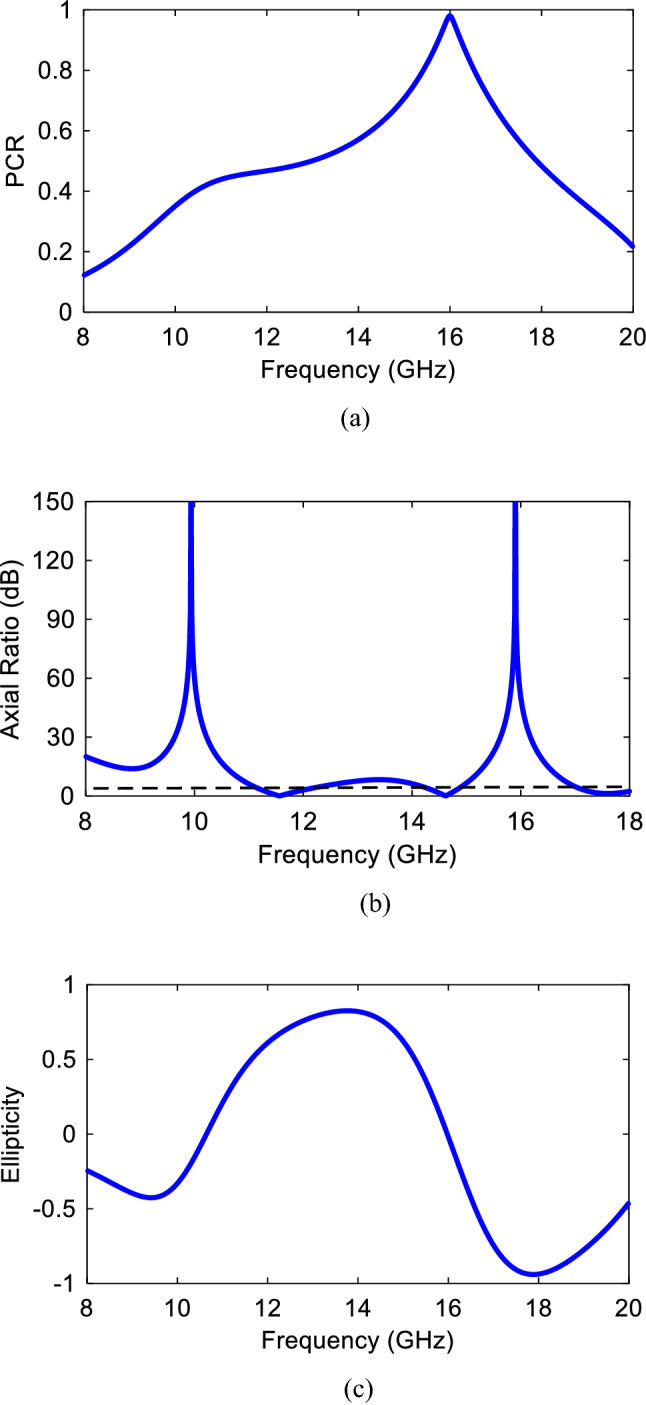
(**a**) Polarization converter ratio, (**b**) axial ratio, (**c**) ellipticity.

To verify, Fig. 2 shows the magnitude and phase results of reflection characteristics when the structure is illuminated by a normal incident wave. Clearly, it can be found that the magnitude is higher than 0.8 in the frequency band of 15.5–16.5 GHz and unity value at 16 GHz. The substantial polarization conversion property of the presented structure can be attributed to strong plasmonic resonance generated at 16 GHz. It means that $\left|{r}_{xy}\right|=1, \left|{r}_{yy}\right|=0$. Hence, the illuminated *y*-polarization is rotated to *x*-polarized wave. According to conservation energy theory, expression ${\left|{r}_{yy}\right|}^{2}+{\left|{r}_{xy}\right|}^{2}=1$ should be satisfied. Moreover, the magnitude of co-reflection wave is very small at the resonance frequency (16 GHz). In addition to amplitude results, phase delay caused by anisotropic feature of the structure is shown in Fig. 2b. It should be noted that the phase feature is only simulated for *y*-polarized incident wave and the similar phase diagram can be plotted for the *x*-polarized incident wave. To evaluate the cross-polarization conversion functionality, Polarization Conversion Ratio (PCR) is defined as ${\text{PCR}}={r}_{xy}^{2}/{r}_{xy}^{2}+{r}_{yy}^{2}$ for reflection mode which is the ratio of the power reflected in the cross-polarized component to the total reflected power. It is obvious from PCR results shown in Fig. 3a that, the PCR value is more than 0.8 within cross-polarization rotation band of 15.5–16.5 GHz with maximum value of unity at the resonance frequency of 16 GHz confirming the complete conversion. Moreover, PCR value is more than 0.5 from 14.3 to 17.3 GHz indicating that more than half of the energy is converted to its orthogonal counterpart in this band with maximum at 16 GHz. A comprehensive study is required to figure out the linear-to-circular polarization functionality, amplitude and phase parameters of mutually orthogonal fields. For this purpose, the amplitudes should be the same and odd multiples of $\pm \frac{\pi }{2}$ should be considered for phase difference of the orthogonally fields. In the next step, to evaluate the polarization state of the reflected linearly and especially circularly polarized wave, the axial ratio is presented in Fig. 3b. Here, according to^31^ the AR value is defined and calculated by:

2a $$AR=20{log}_{10}R$$

2b $$R=\frac{\left({E}_{x}^{r}/{E}_{y}^{r}\right){\mathit{cos}}^{2}\left(\tau \right)+{\text{sin}}\left(2\tau \right){{\text{cos}}}\left({\Delta \varphi }_{xy}\right)+\left({E}_{x}^{r}/{E}_{y}^{r}\right){\text{sin}}^{2}(\tau )}{\left({E}_{x}^{r}/{E}_{y}^{r}\right){\mathit{sin}}^{2}\left(\tau \right)-{\text{sin}}\left(2\tau \right){{\text{cos}}}\left({\Delta \varphi }_{xy}\right)+\left({E}_{x}^{r}/{E}_{y}^{r}\right){{\text{cos}}}^{2}(\tau )}$$

2c $${\text{tan}}\left(2\tau \right)=\frac{2(\left|{E}_{x}^{r}\right|{\text{exp}}(-j{\varphi }_{x})(\left|{E}_{y}^{r}\right|{\text{exp}}(-j{\varphi }_{y})}{{\left(\left|{E}_{x}^{r}\right|{\text{exp}}(-j{\varphi }_{x})\right)}^{2}{\left(\left|{E}_{y}^{r}\right|{\text{exp}}(-j{\varphi }_{y})\right)}^{2}}{\text{cos}}({\Delta \varphi }_{xy})$$

where $\tau$ is the polarization ellipse angle. It can be found that the criterion for AR < 3 dB is satisfied over two frequency bands: 13 GHz and 18 GHz. Additionally, the AR value is very high at 16 GHz indicating the linear polarization conversion. To get insight the handedness validation of circular polarization, Stokes’ parameters ^37^ are utilized as summarized in Eqs. (3a–3d):

3a $${S}_{0}={\left|{R}_{yy}\right|}^{2}+{\left|{R}_{xy}\right|}^{2}$$

3b $${S}_{1}={\left|{R}_{yy}\right|}^{2}-{\left|{R}_{xy}\right|}^{2}$$

3c $${S}_{2}=2\left|{R}_{yy}\right|\left|{R}_{xy}\right|cos\Delta \phi $$

3d $${S}_{3}=2\left|{R}_{yy}\right|\left|{R}_{xy}\right|sin\Delta \phi $$

According to (3a–3d), normalized ellipticity can be defined as $e={S}_{3}/{S}_{0}$. Clearly, it can be found from Stokes’ parameters that normalized ellipticity of + 1 and − 1 should be satisfied in order to get right- and left-handed circular polarizations (RHCP and LHCP), respectively. Confirmed through Fig. 3c, the ellipticity is almost + 1 at the frequency of 13 GHz. Thus, the RHCP reflected EM wave is produced at that frequency. Accordingly, *y*-polarized incident wave is converted to LHCP at 18 GHz.

### Parametric study

To get a better insight about how different parameters affect the structure, some effective parameters have been investigated for parametric study.

### Variation in ‘*r*’ and ‘*d*’

**Figure 4:**
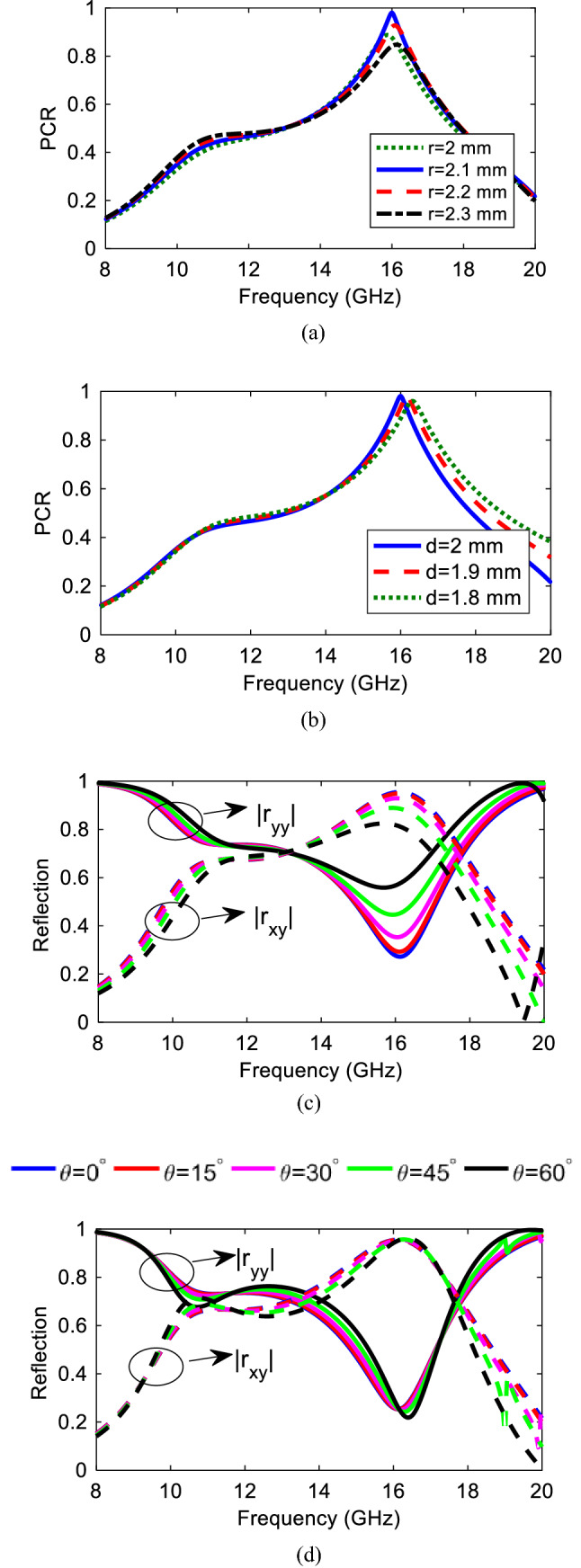
PCR variation for different (**a**) ‘*r*’ values, (**b**) ‘*d*’ values, Magnitude of the Co- and Cross-polarized wave for oblique incident wave in (**c**) *x–z* and (**d**) *y–z* planes.

In order to better understand the cross-polarization conversion, parametric analysis have been conducted in the case of a normal incidence. Apparently, from Fig. 4a the conversion bandwidth is affected by the radius such that by increasing *r* from 2 to 2.2 mm, good polarization conversion occurs in the frequency band of 15.5–16.5 GHz with where PCR reaches to unity at 16 GHz confirming the complete conversion at this frequency. Moreover, from the surface current distribution perspective, increasing *r* to 2.2 mm produces a uniform current on the 45° rotated strip. After that, by increasing the radius from 2.2 to 2.3 mm and more, the structure almost loses its complete conversion such that in 2.3 mm conversion reduces by 20%. This can be explained in this way that by *r* increment, the 45° strip becomes narrow at the center until the surface current decreases significantly and meanwhile increases on the two top and bottom corners of the strip. This also intensifies the current strength between two corners and SRR, generating a larger capacitance as well. The similar analysis is true about the effect of the horizontal arm width of the SRR, (*d*), on the structure as illustrated in Fig. 4b. Additionally, increasing *d* makes the structure larger electrically. At the same time, increasing *d* makes the gap smaller, strengthening the capacitance, equal to more electric charge aggregation, and increasing the inductance as well leading to frequency shift to the lower bands since $f\propto \frac{1}{\sqrt{LC}}$. Therefore, considering both frequency shifts and polarization conversion ratio the best values have been selected as *r* = 2.1 mm and *d* = 2 mm.

### Theta (*θ*) variation

Actually, these structures may be impinged by an arbitrary incident wave angle not necessarily by a normal incidence more specifically in microwave frequencies. Therefore, it is of great importance for a metasurface to show robust respond when illuminated by different angle waves. Accordingly, for a metasurface to show angular stability against oblique incidences it is necessary to be small enough compared to the operating wavelength^31^. Hence, for a transverse electric and magnetic polarizations a complete study is conducted to see how the proposed structure responds to the oblique incidences in a frequency range of 8–20 GHz. It is noteworthy that TE and TM polarizations are defined when the incident electric field is in the *yz*- and *xz*-planes, respectively. It can be seen from Fig. 4c,d that, for both TM and TE polarizations, the magnitude of the cross-polarized reflection coefficient remains stable against the oblique incidence angle up to 60°in the frequency range of 8–19 GHz.

### Theoretical analysis

**Figure 5:**
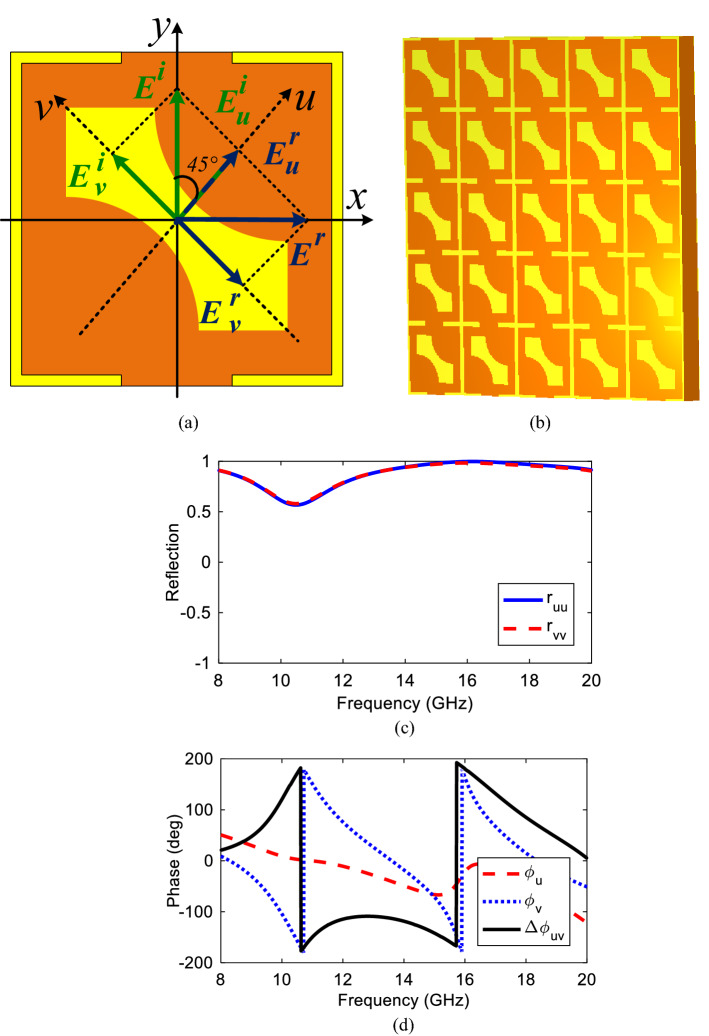
(**a**) The proposed unitcell in new coordinate system, (**b**) 5 × 5 array, (**c**) reflection magnitude, (**d**) phase and phase difference.

To study linear-to-linear conversion mechanism, the *y*-polarized incident wave has been decomposed into two orthogonal components along *u* and *v* axis which makes 45° angle with *y*-axis as shown in Fig. 5a and a 5 × 5 array in Fig. 5b. When reflected components of the incident *y*-polarized wave along *u* and *v* axis compose together, it leads to cross polarization conversion and finally results in *x*-polarized reflected wave. The similar analysis is true for a *x*-polarized incident wave. As expressed in^31^, we can write:

4a $${E}_{y}^{i}=\frac{\sqrt{2}}{2}\left|{E}_{y}^{i}\right|{\text{exp}}\left(jkz\right)\overrightarrow{{e}_{u}}+\frac{\sqrt{2}}{2}\left|{E}_{y}^{i}\right|{\text{exp}}\left(jkz\right)\overrightarrow{{e}_{v}}$$

4b $${E}_{total}^{r}=\frac{\sqrt{2}}{2}\left|{\Gamma }_{u}\right|\left|{E}_{y}^{i}\right|{\text{exp}}\left(-jkz+{\varphi }_{u}\right)\overrightarrow{{e}_{u}}+\frac{\sqrt{2}}{2}\left|{\Gamma }_{v}\right|\left|{E}_{y}^{i}\right|{\text{exp}}\left(-jkz+{\varphi }_{v}\right)\overrightarrow{{e}_{v}}$$

In the case of the structure backed with a metal plate and by ignoring the dielectric losses, $\left|{\Gamma }_{u}\right|\cong \left|{\Gamma }_{v}\right|$.

The co- and cross-polarization magnitude, their reflection phases and phase difference under *u* and *v* axis are determined as *u*-polarized (*r*_*uu*_), *v*-polarized (*r*_*vv*_), ${\varphi }_{u}$, ${\varphi }_{v}$ and $\left|{\varphi }_{v}-{\varphi }_{u}\right|$ as depicted in Fig. 5c,d. Clearly seen, 180° phase difference is achieved in a frequency range of 15.5–16.5 GHz verifying cross polarization conversion with the best conversion state at 16 GHz. Generally, when a linearly polarized EM wave incidents on the structure, the reflected wave includes co- and cross-polarized waves in which the relation between incident and reflected waves can be defined by reflection matrix *R*_*L*_ as in^31^:

5 $$\left(\begin{array}{l}{E}_{x}^{r}\\ {E}_{y}^{r}\end{array}\right)=\left(\begin{array}{ll}{r}_{xx}& {r}_{xy}\\ {r}_{yx}& {r}_{yy}\end{array}\right)\left(\begin{array}{l}{E}_{x}^{i}\\ {E}_{y}^{i}\end{array}\right)={R}_{L}\left(\begin{array}{l}{E}_{x}^{i}\\ {E}_{y}^{i}\end{array}\right)$$

where *R*_*L*_ means linearly polarized reflection matrix, ${r}_{xx}$ and ${r}_{yy}$ correspond to co-polarized reflected fields while ${r}_{xy}$ and ${r}_{yx}$ are to show cross-polarized ones.

Three steps have been considered in this design.

**Figure 6:**
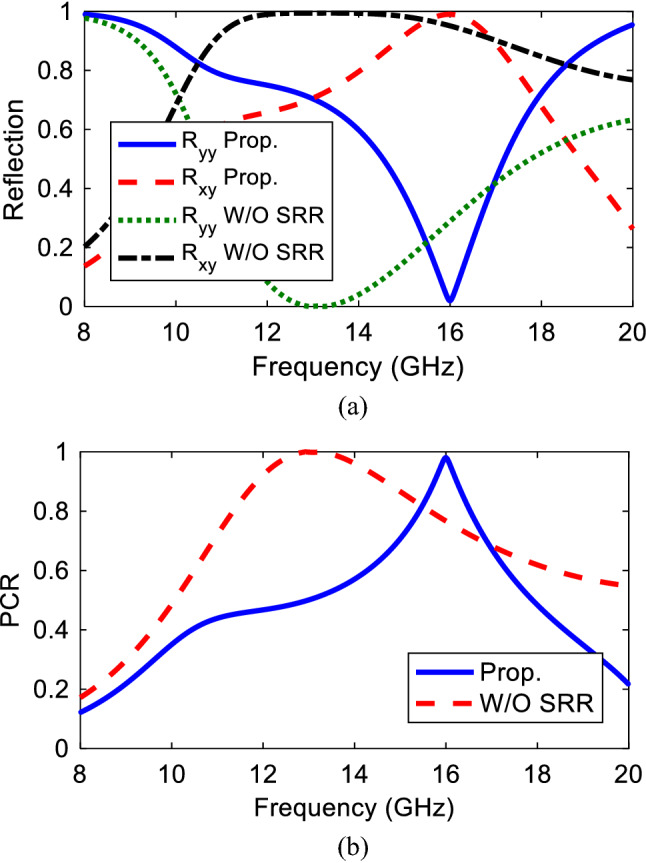
Structure with and without SRR (**a**) reflection magnitude, (**b**) PCR.

Step (1) design of a square patch with two curves cut on two corners, step (2) adding the Split Ring Resonator to the structure and step (3) parametric study to select the final values. In the first step the main purpose is to obtain a cross polarization and according to the PCR value, this can be reached between 15.5 and 16.5 GHz frequency band where the maximum value of unity occurs at 16 GHz. So the *x/y*-polarized incident wave can be successfully transformed to its orthogonal counterpart with complete conversion at 16 GHz. In the second step, a SRR has been added around the structure to disturb the surface current distribution which finally leads to right- and left-handed circular polarization conversion at two frequencies of 13 GHz and 18 GHz, respectively. The co- and cross-reflection magnitudes and PCR for the proposed structure with and without SRR are illustrated in Fig. 6.

### Surface current distribution discussion

To figure out the process of polarization conversion, the working principles behind the conversion mechanism helps more. As a result of various interactions between meta-atoms and incident wave fields, electrically and magnetically polarized meta-atoms forms, which influentially leads to electric and magnetic dipole moments where they can be coupled to both electric and magnetic fields due to the bi-anisotropy nature of the SRR. Equation (6) depicts the relation between the incident fields and spatially averaged effective dipole moments:

6 $$\left[\begin{array}{l}p\\ m\end{array}\right]=\left[\begin{array}{ll}{\text{P}}_{ee}& {\text{P}}_{em}\\ {\text{P}}_{me}& {\text{P}}_{mm}\end{array}\right]\left[\begin{array}{l}E\\ H\end{array}\right]$$

where $$p=\left[\begin{array}{l}{p}_{x}\\ {p}_{y}\end{array}\right]$$ and $$m=\left[\begin{array}{l}{m}_{x}\\ {m}_{y}\end{array}\right]$$ are electric and magnetic dipole moments and $$E=\left[\begin{array}{l}{E}_{x}\\ {E}_{y}\end{array}\right]$$, $$H=\left[\begin{array}{l}{H}_{x}\\ {H}_{y}\end{array}\right]$$ represent electric and magnetic fields while ${\text{P}}_{em}$ stands for electric–magnetic polarizability. The effective surface impedance can be defined by use of electric and magnetic dipole moments of the meta-atoms, given by ${Z}_{s}(\omega ) =\sqrt{{\mu }_{s}(\omega )/{\varepsilon }_{s}(\omega )}$, where ${\mu }_{s}(\omega )$ and ${\varepsilon }_{s}(\omega )$ are frequency-dependent magnetic permeability and electric permittivity, respectively. Furthermore, the frequency dependent reflection coefficient $R(\omega )$ can be determined by the surface impedance in a case of normal incidence as in Eq. (7):

7 $$R\left(\omega \right)=\frac{{Z}_{s}\left(\omega \right)-{Z}_{\circ }}{{Z}_{s}\left(\omega \right)+{Z}_{\circ }}$$

in which ${Z}_{\circ }=120\pi \Omega$ is the impedance of the free space. Equation (7) indicates that, $R = 1$ when the surface impedance of the metasurface is much larger than the free space impedance, ${Z}_{s}({\omega }_{r}) \gg {Z}_{\circ }$, where ${\omega }_{r}$ is the resonance frequency. At such condition, the structure acts as High Impedance Surface, (HIS), at specific frequencies reflecting the incident waves in phase with unity magnitude unlike the out of phase reversal in common reflectors. Based on what has been said before, when two orthogonal components of an incident field are reflected with 0°and 180° phase, polarization plane of the wave is rotated by 90° which results in cross conversion. This implies that the structure behaves as HIS for one of the components while as a common reflector for the other one. To investigate the above-mentioned discussion about the proposed structure, analysis of the surface current produced by the time varying dipole moments are needed which are induced by time harmonic electric and magnetic incident waves. This relationship is explained by Eq. (8) as follows:

8 $$\left[\begin{array}{l}{j}_{s}\\ {M}_{s}\end{array}\right]=i\omega \left[\begin{array}{ll}{\text{P}}_{ee}& {\text{P}}_{em}\\ {\text{P}}_{me}& {\text{P}}_{mm}\end{array}\right]\left[\begin{array}{l}E\\ H\end{array}\right]$$

**Figure 7:**
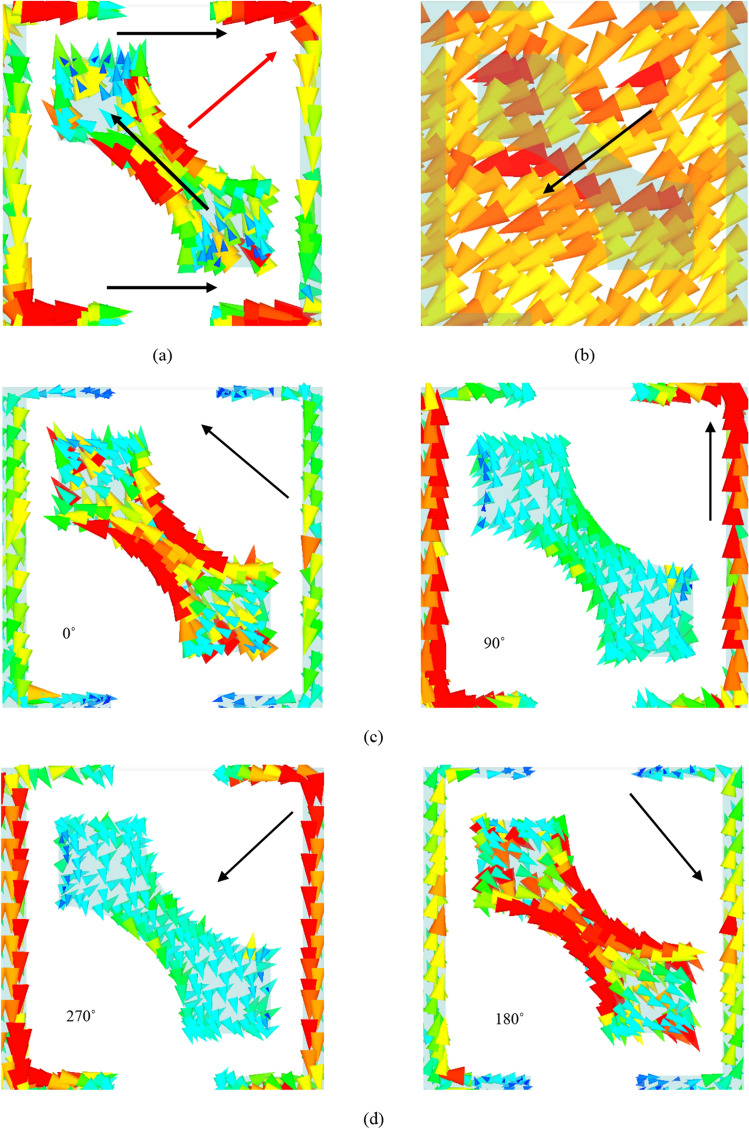

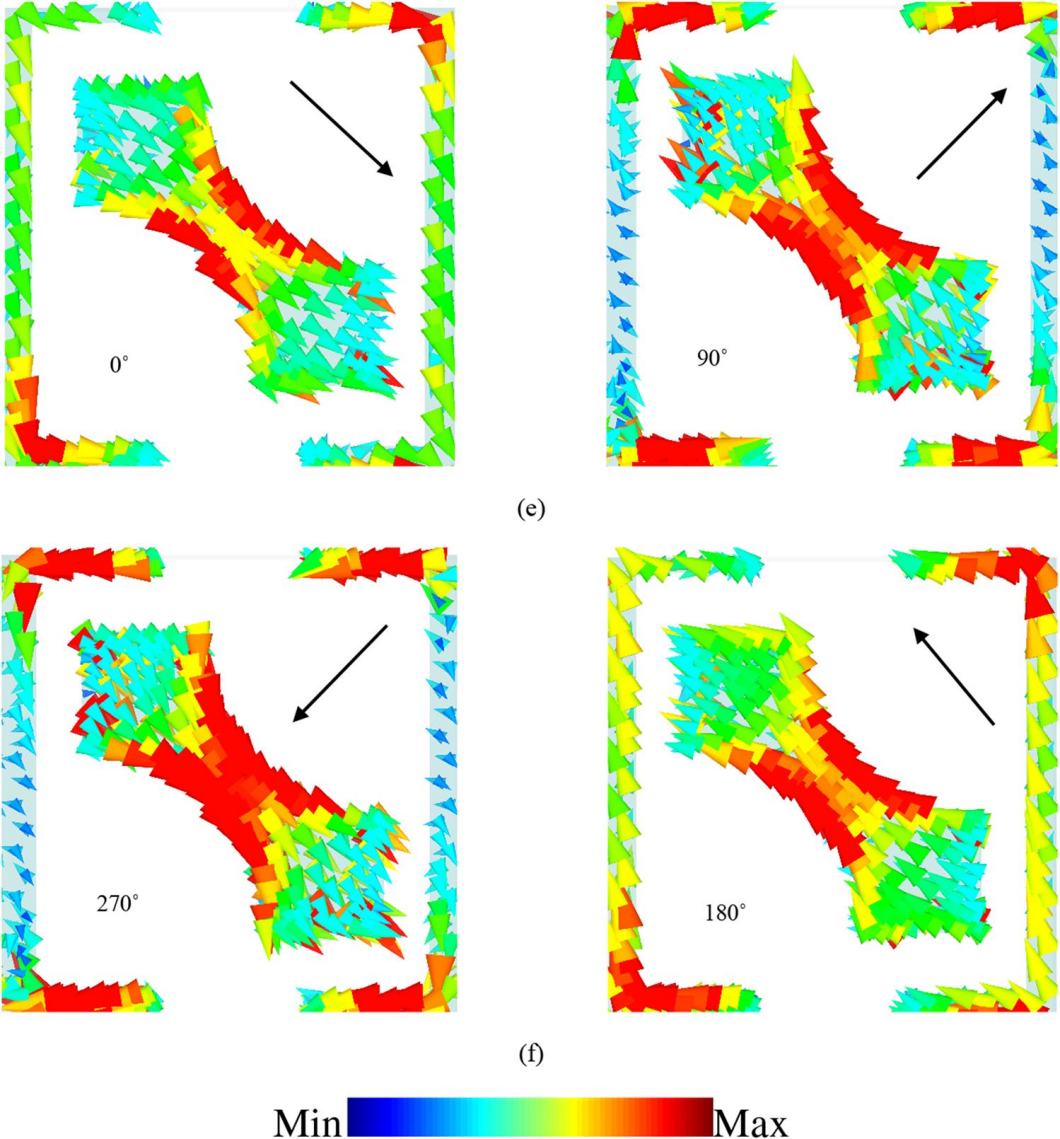
Surface current distribution on (**a**) top and (**b**) bottom of the proposed structure at the frequency of 16 GHz. (Red arrow is the resultant vector of the black arrows.). Surface current distribution at (**c**,**d**) 13 GHz, (**e**,**f**) 18 GHz. (All the figures are generated with CST Microwave Studio software version 2018).

Figure 7 depicts the simulated surface current distribution for resonance frequency of 16 GHz in which linear-to-linear conversion occurs effectively. Figure 7a shows the surface current distribution on the top of the structure at 16 GHz. As can be seen, part of the surface vectors is flowed on the 45° rotated strip and the other part on the SRR (shown in black arrows). The resultant vector which is vector sum of the other two vectors is shown in red arrow. This manifestly reveals that the resonance occurring at 16 GHz is magnetic in nature since the surface current vectors on the top and ground plane are anti-parallel, intensifying the magnetic field in the dielectric substrate. The vector sum of currents is shown in black arrow in Fig. 7. This can be explained in this way that the effective magnetic permeability increases as magnetic flux intensifies, leading to much larger surface impedance compared with free space which finally satisfies the HIS condition, ${Z}_{s}({\omega }_{r}) \gg {Z}_{\circ }$. This results in phase reflection coefficient with unity magnitude and therefore, this leads to change the current flow toward the *x*-direction due to the phenomenon of impedance imbalance along the *y*-direction. Finally, 90° polarization rotation is provided and the *x*-directed polarized wave will be reflected from the surface ^25^. Moreover, Fig. 7c,d and e,f shows the linear-to-circular conversion mechanism at 13 GHz and 18 GHz, respectively. At 13 GHz, Fig. 7c,d shows surface current distribution for 0°, 90°, 180° and 270°, respectively and according to the vectors directions which move clockwise, it can be concluded that right-handed circular polarization conversion happens. Similarly, at 18 GHz this surface current vectors rotations are counter clockwise resulting in left-hand circular polarization conversion as well.

### Equivalent circuit modeling

**Figure 8:**
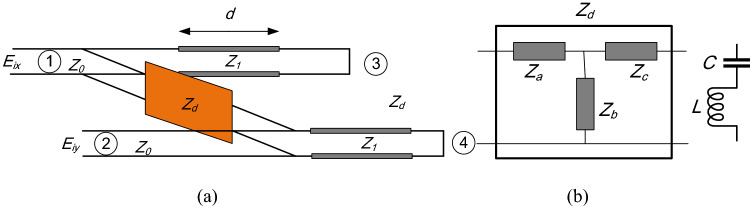
(**a**) The equivalent circuit of the unit cell in Fig. 1. (**b**) T-type of the canonical topology of the two-port interconnection network, Z_d_.

To get insight about the conversion mechanism, according to^38^, a four ports network is applied as shown in Fig. 8a. It is worth mentioning that the main parameters to evaluate the viability of an equivalent circuit to describe a certain structure are: the bandwidth within the circuit can accurately predict the electrical behavior of the structure, the number of elements used, the topology of the circuit and the type of elements used (dependent or independent on the frequency). Another important parameter is the type of excitation and the number of modes that the circuit is able to consider, as some circuits of PPSs are proposed just for normal incidence or for oblique incidence in the main planes. In this regard, the Foster’s circuits present certain properties that make them interesting as an additional tool for the designer together with the aforementioned circuits reported in the state of the art. On the one hand, a Foster’s form does not represent every phenomenological energetic interchange in the structure, but they are encompassed in lumped elements that are frequency-independent. Thus, the resulting circuit is the simplest in terms of number of elements^38^. On the other hand, a Foster’s circuit presents a fixed topology (T, Pi, lattice, etc.), which have been widely studied in the classic circuit theory in a multitude of design strategies. Bi-mode Foster’s equivalent circuit of 2-D with no restrictions on the symmetry of the geometry is presented. The proposed 4-port network shows an invariant circuit topology to the geometry, and is completely made up of invariant-frequency lumped elements independently of the medium used^39^. S_11_ or S_22_ indicate co-polarized while S_21_ or S_12_ is to define the cross-polarized reflection coefficients, respectively under normal incidence of *x*- or *y*-polarized wave propagating along z-axis. Port 3 and port 4 are loaded by conducting short because of the ground plane. Free space impedance is defined as Z_0_ = 377 Ω. To model the FR4 substrate, a transmission line is applied with length $h$ and impedance characteristics of and ${Z}_{s}=\frac{{Z}_{0}}{\sqrt{{\varepsilon }_{r}}}$ is used. Regarding to Fig. 8b, T-type equivalent circuit form is utilized as two transmission lines interconnected by a purely reactive ${Z}_{d}$^38^. Therefore, ${Z}_{d}$ is defined as:

9 $${Z}_{d}=\left[\begin{array}{ll}{Z}_{11}& {Z}_{12}\\ {Z}_{21}& {Z}_{22}\end{array}\right]=\left[\begin{array}{ll}{Z}_{a}+{Z}_{b}& {Z}_{b}\\ {Z}_{b}& {Z}_{a}+{Z}_{c}\end{array}\right]$$

Also, in order to calculate the circuit elements values needed for **Z**_**d**_, the conventional relation between **Z** and **S** matrices are used in Eq. (10). These equations yield to the next matrix distribution for the impedance of the 4-port network:

10 $$Z={\eta }_{0}{\left[I-S\right]}^{-1}\cdot [I+S]$$

**Figure 9:**
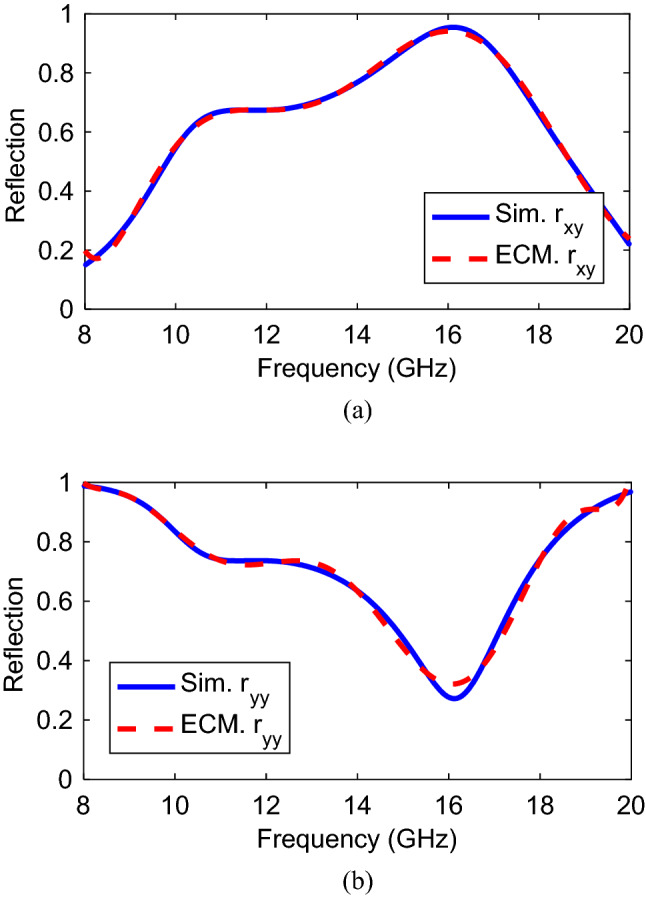
Simulated and equivalent circuit modelling of (**a**) Cross- and (**b**) Co-polarized reflection coefficient for *y*-polarized incident wave.

Based on the Foster representation and for simplicity, a single series resonator (with capacitance C and inductance L) is applied in Fig. 8b to describe the electrical behavior. It should be noted that the obtained reactance from (9) have negative values therefore, the equivalent circuit for impedances ${Z}_{a}$ and ${Z}_{b}$ can be chosen as series inductance and capacitance^38^. In the next step, Advanced Design System, (ADS), is utilized in order to calculate the values of lumped elements. Accordingly, simulated and calculated reflection coefficients for a linearly polarized incident wave is presented in Fig. 9a,b. These figures demonstrate a desired agreement simulation and calculation results. Moreover, the co-polarization coefficients are equal at 16 GHz and the value is − 0.8 dB. The final synthetic values of inductance and capacitance are as follows: C_a_ = 1.02 pF, L_a_ = 3.2 nH, C_b_ = 3.26 pF, L_b_ = 5.31 nH, C_c_ = 2.3 pF, L_c_ = 3.31 nH.

## Measurement results

**Figure 10:**
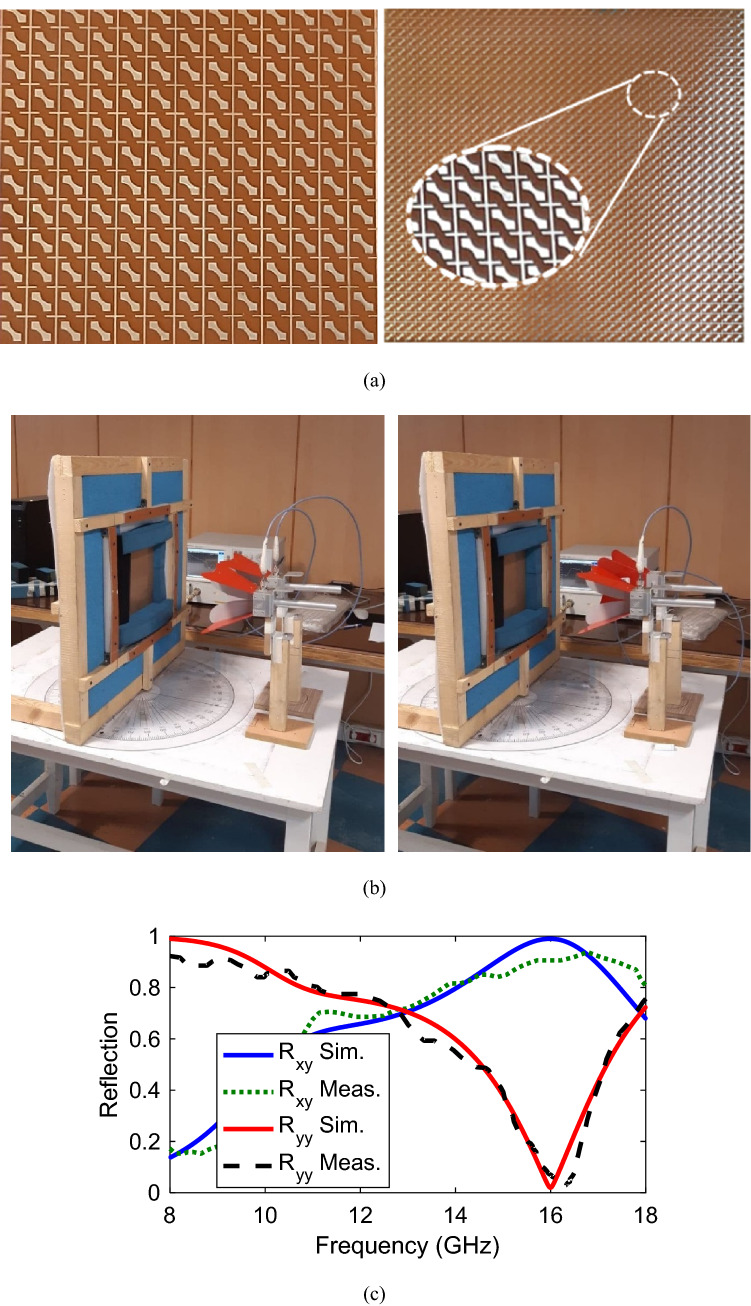
(**a**) Fabricated prototype with overall size of 204 × 204 mm^2^, (**b**) measurement setup for R_yy_ and R_xy_, (**c**) Simulated and measured reflection coefficients.

To confirm the simulation validity, a sample structure consisting of 34 × 34 unitcells has been fabricated with a total size of 204 mm × 204 mm × 2 mm as shown in Fig. 10a and then measured in the Northwest Antenna and Microwave Laboratory. The measurement setups for the fabricated structure have been illustrated in Fig. 10b. Two linearly polarized standard horn antennas (1–18 GHz) which are placed at the same height, have been utilized and connected to the network analyser for transmitting and reflecting. One of the horn antennas is responsible to emit vertical polarized waves, reflected by the structure, and the other one receives both vertical and horizontal. The fabricated structure surrounded by absorbing materials has been placed in front of the horns. The simulation and normalized measured results are shown in Fig. 10c, confirming simulation and calculation results. Horn misalignments and environment noises are some reasons for slight discrepancies between simulation and measurement results.

### Comparison

A comparison has been made among the proposed structure and similar literatures as tabulated in Table 1. Apparently in^14,19^, wide bandwidth is obtained through double layers and via connections. Additionally, except for^30^ with 0.8 mm and^25^ with 2 mm thickness, all the structures have more than 2 mm thickness and larger sizes. The multifunctional property is obtained in^29,31,33^ with a larger unitcell size using only surface current distribution analysis. Two analysis method is utilized in^20^ to realize multifunctional property but with a larger and thicker unitcell. Moreover, in^33^ a multi-band and multi-functional converter with larger size and only surface current distribution is reported recently with wider angular stability. Therefore, compared with others the proposed structure has smaller size, less thickness and at the same time a simple structure. Wide angular stability, (up to 60°), of this work can be a superior feature when compared to similar works. Unlike some of the literatures, more than one theoretical analysis has been used to clarify the structure performance. Finally, linear-to-linear and linear-to-circular (RHCP and LHCP) conversions at three different frequencies have been attained through a simple design.

**Table 1:** Comparison between the proposed structure and similar literatures.

Refs.	Unitcell size (mm)	Sub. thickness (mm)	Operational bandwidth (GHz)	Polarization conversion property	Method of analysis
^14^	8	3.175	5.4–22	Linear-to linear	Surface current distribution
^16^	9	3	6.9–15.4	Linear-to linear	Surface current distribution Destructive interference theory
^19^	14	3.083	5.35–6.3	Circular-to-linear	Surface current distribution ECM
^20^	12	3.6	4.34–4.98, 6.77–6.97, 8.25–8.69 10.72–15.56	Linear-to-linear Linear-to-circular	Surface current distribution reflection matrix
^22^	10	3	6.91–14.31	Linear-to-linear	Surface current distribution
^23^	9.1	3.5	7–19.5	Linear-to-linear	Surface current distribution
^7^	8	3	8.2–23	Linear-to-linear	Surface current distribution Transfer matrix
^24^	5.43	2.5	10.21–24.97	Linear-to-circular	Surface current distribution
^25^	12	2	10.2–20.5	Linear-to-linear Circular-to-circular	surface current distribution Equivalent circuit Equivalent impedance surface by TMM
^29^	7	1.6	8–11 7.5–7.7/11.5–11.9	Circular-to-circular Linear-to-circular	Surface current distribution
^30^	8	0.8	12.4–27.96	Linear-to-linear	Surface current distribution
^31^	8.1	3.1	6.53–12.07 13.7–15.6	Linear-to-linear Linear-to-circular	Surface current distribution
^33^	8.25	2.4	5.3–5.4/7.2–8/12.3–13.76 5.1–5.2/5.6–6.85/8.8–11.2/14.9–20.2	Linear-to-linear Linear-to-circular	Surface current distribution
This work	6	2	15.5–16.5 13, 18	Linear-to-linear Linear-to-circular	Surface current distribution Bi-mode foster circuit model

## Conclusion

A thin, simple and single layer reflective polarization converter metasurface was proposed in this paper to provide multifunctional property. The structure consists of a square with two curves on the top right and bottom left corners rotated 45° with respect to the *y*-axis to realize cross conversion at 15.5–16.5 GHz where, more than 80% conversion ratio achieved with complete conversion at 16 GHz. Moreover, a square SRR around the structure provides linear-to-circular, (RHCP and LHCP) conversion at two frequencies of 13 GHz and 18 GHz, respectively. Bi-Mode Foster and surface current distribution theoretical analysis along with full parametric study were conducted to validate the design accuracy. Additionally, the robustness of the structure to the oblique incident wave is simulated and discussed which indicates an angular stability up to 60°. To verify the numerical and simulated results, a prototype was fabricated and tested and desired agreements obtained as well.
